# Clinical Interpretations of Patient Experience in a Trial of Psilocybin-Assisted Psychotherapy for Alcohol Use Disorder

**DOI:** 10.3389/fphar.2018.00100

**Published:** 2018-02-20

**Authors:** Michael P. Bogenschutz, Samantha K. Podrebarac, Jessie H. Duane, Sean S. Amegadzie, Tara C. Malone, Lindsey T. Owens, Stephen Ross, Sarah E. Mennenga

**Affiliations:** Department of Psychiatry, New York University School of Medicine, New York, NY, United States

**Keywords:** addiction, alcohol use disorder, psilocybin, psychedelics, hallucinogen

## Abstract

After a hiatus of some 40 years, clinical research has resumed on the use of classic hallucinogens to treat addiction. Following completion of a small open-label feasibility study, we are currently conducting a double-blind placebo-controlled clinical trial of psilocybin-assisted treatment of alcohol use disorder. Although treatment effects cannot be analyzed until the study is complete, descriptive case studies provide a useful window into the therapeutic process of psychedelic-assisted treatment of addiction. Here we describe treatment trajectories of three participants in the ongoing trial to illustrate the range of experiences and persisting effects of psilocybin treatment. Although it is difficult to generalize from a few cases, several qualitative conclusions can be drawn from the data presented here. Although participants often find it difficult to describe much of their psilocybin experience, pivotal moments tend to be individualized, extremely vivid, and memorable. Often, the qualitative content extends beyond the clinical problem that is being addressed. The participants discussed in this paper experienced acute and lasting alterations in their perceptions of self, in the quality of their baseline consciousness, and in their relationship with alcohol and drinking. In these cases, experiences of catharsis, forgiveness, self-compassion, and love were at least as salient as classic mystical content. Finally, feelings of increased “spaciousness” or mindfulness, and increased control over choices and behavior were reported following the drug administration sessions. Ultimately, psilocybin-assisted treatment appears to elicit experiences that are extremely variable, yet seem to meet the particular needs of the individual.

## Introduction

The past decade has seen a renewed interest in the therapeutic potential of psychedelics in psychiatry. A meta-analysis demonstrated that controlled trials of LSD-assisted treatment of alcoholism from the 1960s favored LSD-treated patients over controls, with robust and clinically meaningful effects persisting up to 6 months after a single high-dose LSD session (Krebs and Johansen, 2012). Recently, small non-controlled trials have demonstrated impressive improvements among participants treated with psilocybin for alcohol use disorder (AUD) and cigarette addiction (Johnson et al., 2014; Bogenschutz et al., 2015). Randomized controlled trials are now testing the efficacy of psilocybin-assisted treatment of tobacco, alcohol, and cocaine use disorders. Psilocybin-assisted psychotherapy has also been shown to be effective in the treatment of depression and anxiety in cancer patients (Griffiths et al., 2016; Ross et al., 2016), and an open-label trial showed improvement in treatment resistant depression following psilocybin treatment (Carhart-Harris et al., 2016).

Most clinicians and researchers exploring clinical applications of hallucinogens combine the administration of hallucinogens with psychotherapy, believing that therapeutic benefit derives from the subjective experiences occasioned by the drug, coupled with successful integration of these experiences through psychotherapy. Recent studies of hallucinogen-assisted treatment of addiction have employed no more than three high-dose psilocybin sessions in the context of evidence-based addiction treatment and a ‘container’ of preparation, support and integration for the hallucinogen experiences. These models are similar to the original “psychedelic-peak therapy” models that were utilized in research and clinical practice in the 1950s through early 1970s (Sherwood et al., 1962; Hoffer, 1967; Pahnke et al., 1970; Grof et al., 1973). In contrast, the psychedelic-chemotherapy model used a single high-dose session of psychedelic treatment with minimal psychotherapy (e.g., Hollister et al., 1969), while the psycholytic model employed lower doses of psychedelics across many sessions in the context of psychodynamic psychotherapy (Pahnke et al., 1970).

Historically, the psychedelic therapy model held that ‘peak-psychedelic’ experiences were crucial to therapeutic benefit. The definition of a peak-psychedelic experience is not clear, but literature from the 1950s-early 1970s describes something similar to a mystical experience, characterized primarily by the experience of unity or loss of the sense of a separate self. Mystical experiences have more recently been described as experiences high in unity/oneness internally and with one’s surroundings, insightfulness, knowledge of ultimate reality, and spiritual or religious sacredness (Stace, 1960; Pahnke, 1963; MacLean et al., 2012; Barrett et al., 2015). Recent small studies have supported the hypothesis that mystical experience is a mediator of change (Johnson et al., 2014; Bogenschutz et al., 2015), although more general measures of intensity also predicted improvement in our completed pilot AUD trial (Bogenschutz et al., 2015). In our initial experience with psilocybin-assisted treatment of AUD, we observed that participants reported a wide range of psychological experiences and processes that they considered to be critical to positive changes with respect to their drinking and related domains. Many of these experiences are not adequately captured by the measures that have been used in hallucinogen-assisted treatment trials. Acute effects measures have focused on mystical experiences, sensory/perceptual changes, and dysphoric effects. Persisting effects measures are somewhat broader, but likewise, have not included items that would capture some of the change processes reported by our participants.

Qualitative studies of participants in recent trials of psilocybin-assisted treatment of cancer-related anxiety and depression have provided useful insight into participants’ psilocybin experiences and the therapeutic process (Belser et al., 2017; Swift et al., 2017). However, such qualitative studies have not yet been published for recent trials utilizing psilocybin to treat addiction. Here, we present case reports of three participants in our ongoing randomized controlled trial of psilocybin-assisted treatment of AUD to describe some of the change processes that have been observed among trial participants. Our goal is to describe psychological processes that will inform the design, measures, and hypotheses of future trials.

## Materials and Methods

The patients described here were participants in a randomized controlled trial to evaluate the efficacy of psilocybin-assisted treatment for AUD, relative to diphenhydramine control, and to investigate several possible mechanisms of action. The therapeutic model used in the current double-blind study is based on that used in a completed open-label pilot study (Bogenschutz et al., 2015) which demonstrated a significant improvement in drinking following psilocybin-assisted treatment. The total duration of psychosocial treatment in the double-blind period is 12 weeks, with drug administration sessions occurring at 4 and 8 weeks. In the first medication session, psilocybin 25 mg/70 kg vs. diphenhydramine 50 mg is administered orally. Depending on the response in the first session, the dose for the second session may be increased to psilocybin 30 mg/70 kg or 40 mg/70 kg vs. diphenhydramine 100 mg, or held at the original dose. Following completion of the double-blind period (34 weeks after randomization), all participants who meet interim safety criteria are offered an additional open-label session in which psilocybin is administered (25 mg/70 kg for those who received diphenhydramine in the double-blind sessions, 25–40 mg/70 kg for those who received psilocybin in the double-blind sessions). Drinking outcomes and several potential mediators of treatment effect, including motivation, self-efficacy, craving, depression, anxiety, and spiritual dimensions of the experience are measured at multiple timepoints until 50 weeks after the first drug administration session, for a total of 54 weeks from the initiation of treatment.

The therapy model used in this study has been described in a recent publication (Bogenschutz and Forcehimes, 2016). Therapy sessions focus on the problematic use of alcohol as well as preparation for and integration of the medication sessions. The therapy is conducted by a team of two therapists, one responsible for the alcohol-specific treatment, the other responsible for the hallucinogen-specific treatment. See **Supplementary Materials** for a detailed description of the treatment model used in this trial, and **Supplementary Figure S1** for a diagram of the timeline of the study describing temporal relationships between medication sessions, therapy sessions, and assessments.

Here, we report data collected for the trial, along with qualitative information from audiotapes of therapy sessions and observations of the study team. The information presented in this paper is descriptive and not selected at random. The participants presented here have not been unblinded for this report, so whether they received psilocybin or diphenhydramine in the first two medication sessions is unknown, although it is known that the medication delivered in the optional third dosing session was psilocybin, and not diphenhydramine. Participants provided written informed consent for publication of these de-identified reports. See **Supplementary Materials** for descriptions of the quantitative outcome measures presented.

## Results

**FIGURE 1:**
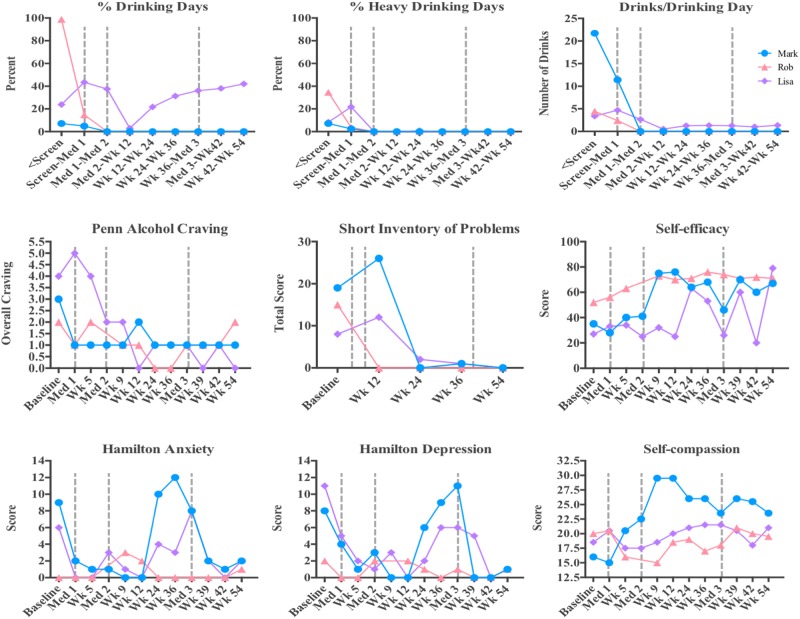
Quantitative data from the three cases presented. Percent drinking days, percent heavy drinking days, and average number of drinks per drinking day are shown for each participant throughout the study. Scores on the Penn Alcohol Craving, Short Inventory of Problems, Self-Efficacy, Hamilton Anxiety, Hamilton Depression, and Self-compassion scales are presented throughout the study.

**Table 1** summarizes demographics and baseline characteristics of the three participants whose experiences are described. **Figure 1** summarizes drinking behavior and other drinking-related self-report measures obtained before and after the medication sessions. **Supplementary Figure S2** illustrates participants’ scores on measures of acute hallucinogen effects collected following each medication session.

**Table 1:** Patient Demographics and pre-treatment history.

Pseudonym	Mark	Rob	Lisa
Age	20’s	40’s	50’s
Sex	Male	Male	Female
Race/Ethnicity	Non-Hispanic/White	Black/African American	Hispanic/Other Latin American
Marital status	Single, never married	Single, never married	Divorced
Education	Bachelor’s degree	Associate degree	Bachelor’s degree
Employment	Full-time	Unemployed	Full-time
Alcohol abuse: age of onset	Early teens	Late teens	Late twenties/Early thirties
Alcohol abuse: past treatment	1x residential alcohol treatment 5x outpatient alcohol treatment 200 AA meetings Regimen of Disulfiram	No prior treatment	1x residential alcohol treatment 1x outpatient psychiatric treatment 29 AA meetings
Other substance use disorders and psychiatric history	None	None	None
Family history of alcohol problems	None	2x first degree relatives: mother and father	2x first degree relatives: mother and father 3x second degree relatives: aunts and uncles
% days abstinent at baseline	93%	1%	76%
Average drinks per drinking day at baseline	22	4	3
Past hallucinogen use	3x MDMA	2x Mescaline	No hallucinogen use

### Mark

Mark was a Caucasian male in his 20s living with his parents and working full-time at the time of enrollment. His binge drinking began in his teens and intensified into adulthood. He reported frequent blackouts and occasional absences from work due to drinking episodes that lasted for days at a time. At baseline, he reported drinking on six of the past 84 days, with an average of 22 drinks per drinking day. Mark had made multiple unsuccessful attempts at treatment, and had attended hundreds of AA meetings. Mark started the study with the intention of attaining complete abstinence from alcohol. He said, “I just want to stop and have a normal life.”

During the first medication session, Mark encountered his anxiety and fears associated with failure. Though the effects were mild and difficult for him to communicate in words, he said, “It was almost like finding the Holy Grail and the answer to all of life’s questions.” Self-report assessments revealed that he had experienced a session of moderately high intensity. In the month that followed, Mark remained abstinent and was surprised at how easy this was and how little he thought about alcohol.

Mark’s second medication session was higher in both dose and intensity. He was confronted by the harmful effects that his drinking had on himself and others. He stated that “at one point, I felt I could have cried for joy,” when realizing that he was being given “a new slate.” In the following weeks, he reported increased motivation and drive as well as a strong desire to contribute to the world in a meaningful way. He said, “I feel like I’m maturing. Maybe a part of me died when I gave up alcohol.”

Mark remained abstinent during the 7 months following the second medication session. He opted to have the third open-label medication session with the hope that it would help with his work-related anxiety. He described the experience as “a crash course” in dealing with feelings of disappointment, regret, shame, and unworthiness. He also reported “a couple of eureka moments,” and said that the session ended with “calmness, comfort, and reassurance.” He said, “I wouldn’t be surprised if I never drank again” and added, “I got exactly what I need out of the experience.” One month after this session, he remained abstinent and expressed a great deal of gratitude for being able to participate in the study. Two years from his initial intake, Mark contacted the study team to report that he continued to remain abstinent.

### Rob

Rob was an African–American male in his 40s who was unemployed at enrollment in the trial. He had been drinking heavily since college, and his alcohol use had caused him to discontinue his studies and his promising athletic career. He was raised in a family that drank heavily, and his father had died due to complications associated with alcoholism. Since the passing of his father, Rob became very concerned about his own health in relation to his own alcohol use. His drinking was also in direct conflict with his Muslim faith. At the time of enrollment he had consumed alcohol on 83 of 84 days, with an average of four drinks per drinking day. He was able to achieve 8 days of sobriety prior to his first medication session.

Rob’s first medication session was dominated by strong nausea and abdominal pain. During the peak effects of the study medication, he sat upright, attempting to vomit, but was only able to spit repeatedly into a wastebasket. In debriefing the following day, he reported briefly sensing the presence of his father and communication of mutual forgiveness. However, upon remembering that his religion did not permit the living to communicate with the dead, he decided to resist the effects of the drug to the best of his ability and began to feel ill. He continued to combat the drug effects for the remainder of the session. At one point, he perceived his saliva in the wastebasket as swirling beer suds and interpreted that as representing the toxic effects of his drinking. He then proceeded to spit out what he interpreted as his shame, resentment, regret, and anger. Eventually he became too exhausted to continue fighting the drug’s effects, at which point he lay down on the couch and gradually began to experience increased comfort.

In subsequent therapy sessions, Rob reported that the medication session had been the most painful experience of his life, commenting that “nothing ever felt worse than those 2 hours.” He was pleased to have “weathered the storm,” and as a result of the “ordeal,” he reported an increased sense of urgency to get his life moving in a positive direction. He acknowledged that he judged himself harshly for not making more of an effort to keep his life on track in the past. However, as he gained confidence from the progress made in his life, he began to feel more forgiving of himself. He was hired at a new job within 4 weeks of the medication session and also enrolled in school. Rob also reported that he valued the moment of contact with his father, and that the session had affirmed and strengthened his resolve to live according to his religious principles. He declined the second and third medication sessions, but completed all other aspects of the therapy and assessments for the study. At his last follow-up visit (54 weeks after beginning the study,) he remained abstinent with little desire to drink and happily reported that he was employed and pursuing a degree in social work.

### Lisa

Lisa was a Latin-American female in her 50s with a family history of alcoholism, physical and emotional abuse, abandonment, and neglect. Her problematic drinking began around the age of thirty and resulted in social isolation, hangovers, strong feelings of guilt and shame, and severe self-critical thoughts. At the start of the study she expressed concern regarding the effects that drinking was having on her physical and mental health. Lisa had made multiple previous attempts at treatment, and attended a total of 29 AA meetings, with the most recent meeting in 1993. At study enrollment, she had been drinking on 20 out of the previous 84 days, averaging three drinks per drinking day.

During the initial session, Lisa spent time exploring her mother’s neglect and abuse, but noted that she did not experience any antagonism toward her. She examined the negative feelings that she harbored for herself and feelings of alienation from God. She remembered an inner voice exclaiming to God, “Why did you leave me?” to which God responded, “Why are you so controlling?” After this session, she noticed a significant brightening of her mood and a lasting decrease in self-critical thinking. In response to God’s message about her controlling tendencies, Lisa chose not to commit herself to complete abstinence at that time, though she found herself drinking much less.

In the second session, Lisa received a higher dose of medication and experienced an amplification of thought moving her into a confused and chaotic state. Underneath the chaotic thinking, she identified a deep well of overwhelming sadness. She was able to eventually surrender control over her thoughts and entered into a state of peacefulness, until her thoughts quieted completely. She heard her own inner voice rupturing the quiet, whispering into her ear: “I’m going to tell you a secret. It’s the worst-kept secret in the universe because everyone knows it but you. You are a perfect creation of the universe.” At that moment she felt that everything in existence was unified and was made of love, though a part of her remained reluctant to fully believe this to be true. The voice repeatedly presented her with this reality, asking “do you believe this?” over and over until each one of her objections had been addressed and dismissed. She examined herself and found that she finally did accept this to be true, which propelled her into a state of profound self-acceptance and wellbeing. She later said, “All there is is love, this is all that you are, this is all that matters.”

Following her medication session, Lisa reported that her self-critical thoughts had dissolved and that alcohol had lost almost all of its appeal. She said that the medication sessions had illuminated how she had been unkind to her body and had been harming herself with alcohol. She noted her ability to manage stress and found that she was making time to care for herself through socialization, relaxation, and a resumed meditation practice. She reported improved concentration, a lack of negative self-talk, decreased anxiety, and a spacious quality of mind, stating that, “the noise can bubble up but it doesn’t overwhelm me. When these little anxieties walk in this big room they seem so little. I feel peaceful, and I feel safe. It feels good to be in my body. I’ve found myself taking wonderful breaths. The negative remarks don’t even pop into my head.”

Lisa elected to participate in the open-label session. Before her third dosing session, she reported a dramatic and sustained increase in her anxiety, which she attributed to the results of the recent presidential election. She reported that the positive effects from the two previous sessions had persisted, and that alcohol was no longer problematic. She described being able to consume an occasional glass of wine while remaining free from the compulsion to overindulge. The only instance of drinking to excess was on one isolated occasion, which was the night of the election. Her intention for the third session was to find relief from her apprehension regarding the election outcome. She described the medication experience as consisting of several hours of pure and intense anxiety, with very little specific thought or perceptual content. The following day she reported that her anxiety had lifted and that she was feeling calm and peaceful. At 54 weeks, Lisa reported a persisting reduction in alcohol consumption and alleviated anxiety.

## Conclusion

These data represent a sampling of themes that have occurred during treatment. The full diversity of patient experiences extend well beyond the scope of the three cases presented here.

Our intention is not to provide support for generalizations about frequency of phenomena occurring during psilocybin-assisted treatment, or the relationship of phenomena to outcomes. Based on the cases presented here, we can draw some general observations about participant experiences.

Primarily, we note that experiences are extremely variable. While some experiences prominently feature elements of the classic mystical or peak-psychedelic experience, others instead center on feelings of forgiveness, self-compassion, and love, as well as catharsis and acceptance of past behavior. The dosing sessions tend to evoke material that is personally meaningful, with content that is uniquely relevant to each individual participant. The content of these medication sessions tended to match the perceived needs of the participant, supporting their efforts to recover from AUD and improve their lives in other ways. Of note in the quantitative data presented, while these three participants entered the study with distinct drinking profiles ranging from infrequent binge drinking to daily alcohol use, each achieved a reduction in alcohol intake reflective of his/her goals.

The medication experiences do not necessarily focus on alcohol to any great extent, and when they do, they may present the issue of drinking in a larger psychological or spiritual context. In addition to changes in drinking, participants have reported marked changes in their perception of self and in the quality of their daily baseline consciousness, including feelings of increased “spaciousness” and ability to calmly attend to the present moment. While trajectories of change in anxiety, depression, and self-efficacy differed, each participant reported increased self-efficacy and decreased craving, alcohol-associated problems, anxiety, and depression by the final timepoint. Lacking a coherent verbalized narrative about the experience (whether due to lack of articulateness/education/vocabulary or the ineffability of the experience) does not appear to preclude the experience from being meaningful or leading to positive change. Although the experiences are difficult to describe, the pivotal moments are often highly vivid and memorable.

Based on these reports, it seems likely that mediators of change range from psychological to mystical. Our participants reported experiences rich in self-compassion, love, connection, catharsis, and psychodynamic material, in addition to mystical content. These experiences were perceived by some as responsible for the improvement participants were able to achieve. Our observations suggest that individual experiences are uniquely suited to facilitate personal growth. These observations support the therapeutic approach used in this study, which helps participants find personal meaning in their experience, without suggesting that certain types of experiences are better than others. It is possible that this model encourages diversity and “personalization” of the experience. We do not yet have empirical evidence to support that our approach is superior to any other. If controlled trials demonstrate that this model is effective for treatment of AUD, additional work will be necessary to determine which therapeutic approaches are most effective, and whether different therapeutic approaches are optimal for different types of patients.

## Ethics Statement

This study was carried out in accordance with the recommendations of the institutional review board of the New York University (NYU) School of Medicine, with written informed consent from all subjects. All subjects gave written informed consent in accordance with the Declaration of Helsinki. The protocol was approved by the institutional review board of the New York University (NYU) School of Medicine.

## Author Contributions

MB is the principal investigator of the parent trial that served as the platform for the data collection presented in this manuscript. MB, JD, and SR acted as study therapists for the parent trial that served as the platform for the data collection presented in this manuscript. SP, SA, TM, LO, and SM acted as study coordinators and performed data collection for the parent trial that served as the platform for the data collection presented in this manuscript. All authors contributed to the conceptualization and writing of this manuscript, and all authors have approved the final version of this manuscript.

## Conflict of Interest Statement

The authors declare that the research was conducted in the absence of any commercial or financial relationships that could be construed as a potential conflict of interest.

## Supplementary Material

FIGURE S1 Overview of study design. Study weeks, treatment components, and corresponding assessment visits are depicted.

FIGURE S2 Measures of Acute Hallucinogen Effects. Scores for each participant on the States of Consciousness Questionnaire/Mystical Experience Questionnaire, the 5-Dimensional Altered States of Consciousness Scale, and the Hallucinogen Rating Scale are shown for each medication session.
