# Supplementary figures and images for: Clinical Interpretations of Patient Experience in a Trial of Psilocybin-Assisted Psychotherapy for Alcohol Use Disorder
Source: Front Pharmacol. 2018 Feb 20;9:100. doi: 10.3389/fphar.2018.00100 (PMC5826237; doi:10.3389/fphar.2018.00100)

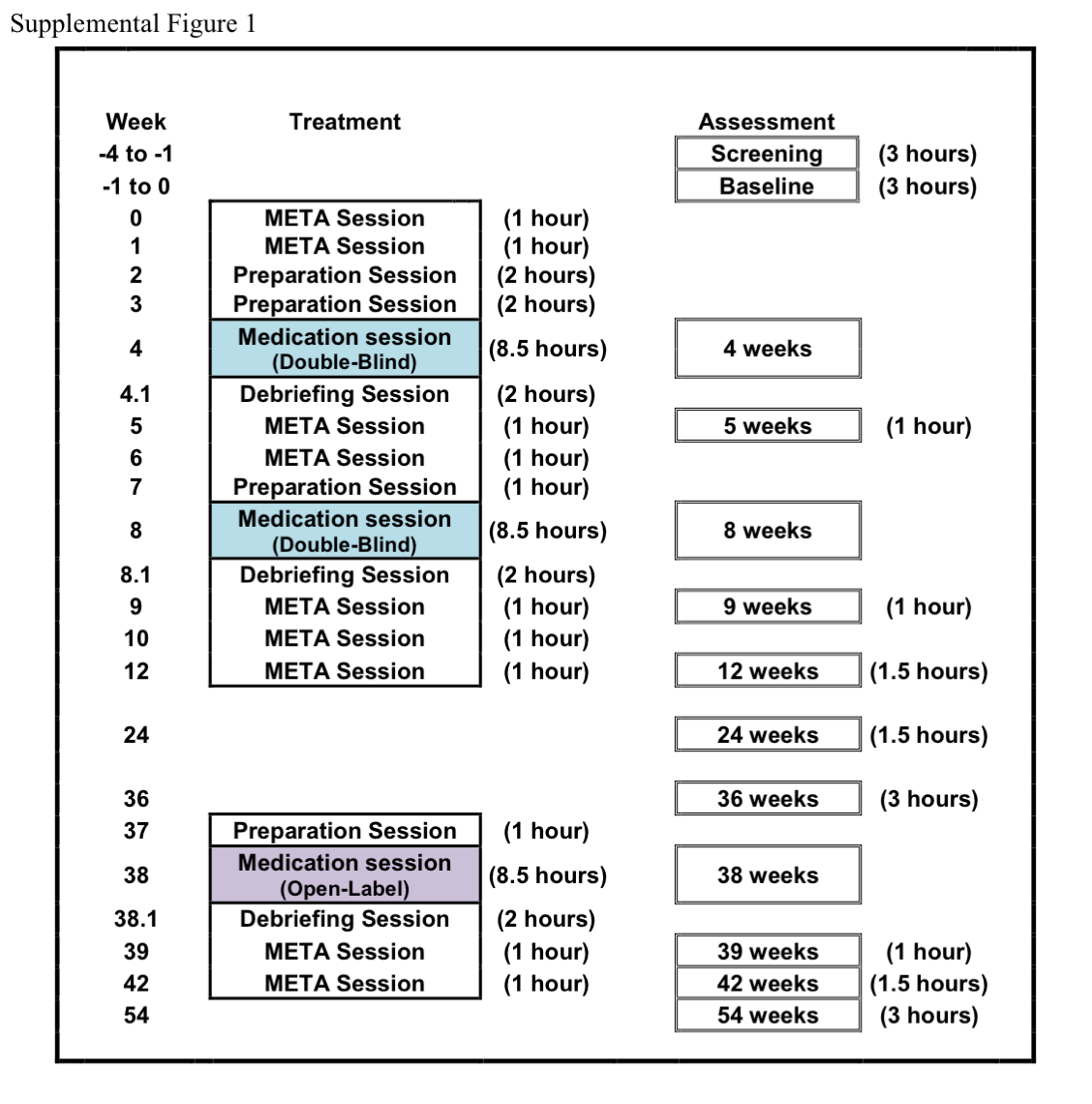

Supplement: FIGURE S1 — Overview of study design. Study weeks, treatment components, and corresponding assessment visits are depicted. [file Image_1.TIFF]

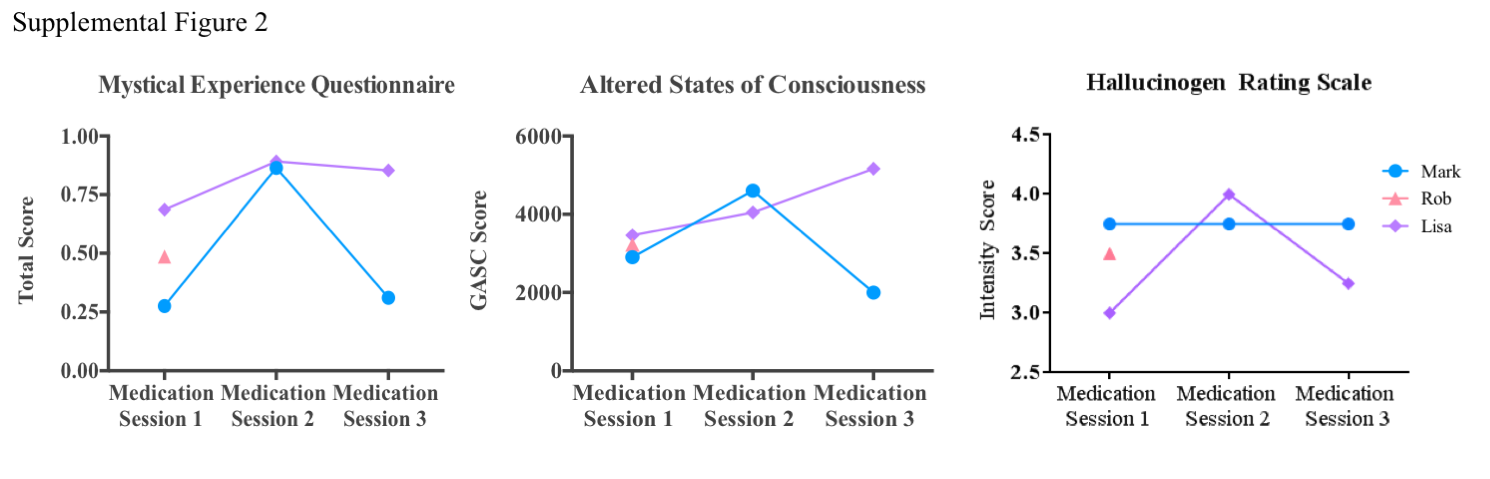

Supplement: FIGURE S2 — Measures of Acute Hallucinogen Effects. Scores for each participant on the States of Consciousness Questionnaire/Mystical Experience Questionnaire, the 5-Dimensional Altered States of Consciousness Scale, and the Hallucinogen Rating Scale are shown for each medication session. [file Image_2.TIFF]
