# Supplementary material for: Clinical Interpretations of Patient Experience in a Trial of Psilocybin-Assisted Psychotherapy for Alcohol Use Disorder
Source: Front Pharmacol. 2018 Feb 20;9:100. doi: 10.3389/fphar.2018.00100 (PMC5826237; doi:10.3389/fphar.2018.00100)
Supplement: Supplementary file 3 [file Supplementary_Material.pdf]

Supplemental Materials

Methods

Therapeutic models of psychedelics

The two prominent therapeutic models born in the 1950s through early 1970s were *psycholytic* and *psychedelic* therapy (Grinspoon & Balakar, 1997; Grof, 2008). In the psycholytic method, low to moderate doses of hallucinogens were administered on multiple occasions to facilitate therapy that was based on traditional psychoanalytic principles, i.e., helping the patient to become aware of unconscious desires, emotions, attachments, and self-representations, and resolving intrapsychic conflicts (Buckman, 1967; Leuner, 1967). Psychotherapy was conducted while the patient was under the influence of the drug. The psychedelic method used higher doses of hallucinogens administered on no more than a few occasions, with the goal of eliciting "peak-psychedelic" or mystical experiences. Such experiences are characterized by unity (sense of oneness), transcendence of the ordinary experience of space and time, sense of sacredness, sense of deep truth or ultimate meaning (noetic quality), deeply felt positive mood, and ineffability (W. N. Pahnke, 1969a). It has been held that such experiences often facilitate lasting change in habitual patterns of thought, behavior, experience of emotion, and even personality (Hoffer, 1967; Sherwood, Stolaroff, & Harman, 1962). Although the psycholytic and psychedelic models are conceptually distinct, some clinicians and investigators used both, or created hybrid models (Grof, 2008; Masters & Houston, 2000).

The therapy model employed in the current study is similar to the psychedelic model, in that it includes no more than 3 high-dose psilocybin sessions embedded within a 'psychosocial package' of evidence-based addiction treatment and preparation, support and integration for the drug administration sessions (Bogenschutz et al., 2015). The therapy is conducted by a team of two therapists, one responsible for the alcohol-specific treatment (META), the other responsible for the hallucinogen-specific treatment (PSI). The approach used in the current study deviates from the traditional psychedelic model in that we do not instruct participants to interact with the

medication-induced state in a way that promotes the possibility of a peak-psychedelic or mystical experience. Rather, we instruct participants to relinquish control of the experience, and suggest to them that whatever experience they have will be the necessary one for their own personal change.

Motivational Enhancement and Taking Action (META)

The META content is largely based on materials used previously in multisite trials (W. R. Miller, 2004; W.R. Miller, Zweben, Diclemente, & Rychtarik, 1992; Obert & Farentinos, 2000). Because the number of sessions indicated is greater than the 3-4 sessions typically used in motivational enhancement therapy (MET), and because of the relatively high motivation for change seen in many of the pilot study participants following the first psilocybin session, the META manual developed for this protocol includes greater emphasis on exploration of a patient's goals for change and the development and implementation of specific strategies to meet those change goals.

The first two META sessions occur during the month prior to the first medication session (psilocybin or diphenhydramine control). In the first session, the therapist uses open motivational interviewing to elicit and clarify the patient's intrinsic motivation for change. During this session, therapists also offer feedback from the baseline assessment, specifically focusing on drinking percentile relative to population norms, consequences of drinking, and motivation for change. The focus of the second session is on eliciting the patient's most important values using a values card-sort and exploring the discrepancy between values and behavior as motivation for change.

The two subsequent META sessions (sessions 3 and 4) follow up on the patient's goals for change and experiences during the first medication session in relation to the key values identified during META session 2. During these sessions the therapist and patient develop a specific treatment plan for the remaining sessions. These sessions use a cognitive behavioral framework, which uses the acronym STORC, for Situations, Thoughts, Organic patterns (i.e., physical sensations and emotions), Responses, and Consequences. At each step in the STORC cycle there are usually a number of things that can be done to promote change. Therapists work

with patients to identify specific components of STORC sequences in which the patient seems to be encountering difficulties. Patients are given a workbook that includes a menu of strategies in each of these domains. For example, the Situational Factors Menu will include modules on how to identify problem situations, monitoring urges, ways to change your environment, how to ask others for help, and how to surround yourself with support; the Organic Patterns Menu includes modules on exercise, mindfulness practice, sleep hygiene, and nutrition. For each change goal, the therapist works with the patient to identify strategies potentially useful in facilitating change, and together they choose strategies that form the basis of an individualized change plan, which provides the structure for the subsequent META sessions.

In contrast to the first four sessions, which are highly structured in that all patients receive similar content on feedback, values exercise, and structuring a treatment plan, the remaining sessions are individualized to the unique needs of each patient. Specific pull-out teaching modules with worksheets to be completed during and between sessions offer structure for therapists in the delivery of this portion of the intervention. This allows therapists to use flexible discretion as to the content of these sessions as the needs of the patient evolve. Activities that may be used during these sessions will include 12-step or other self-help involvement, mindfulness practice, exercise, changes in social network, cognitive behavioral self-help, alternative sources of positive reinforcement that do not involve substance use, or further formal alcohol treatment. The plan for change is revisited and revised as needed during each session, and therapists will reinforce progress and revise the plan as needed in collaboration with the patient. Consistent with the motivational interviewing style, these remaining sessions are also intended to be a time to re-engage the patient, continue discussions on experiences and feelings resulting from the medication sessions, support continuing efforts, and address any barriers to goal achievement.

Preparation, Support, and Integration (PSI)

There are two preparation sessions before the first drug administration session, and one before the second and third drug administration sessions. The primary goals for the first preparation session are to conduct a detailed life review, including information about the participant's history, current situation, personality, relationships, goals, etc., and to facilitate the development of rapport between the participant and the clinicians. The second session includes a review of motivation and expectations for the study, detailed information about the possible physiological and psychological effects of study medication, advice on how to deal with dysphoric reactions to study medication, should they occur, identification of any personally meaningful items that the participant will bring to the session (e.g., images, family photographs, objects of personal or religious significance), discussion of ground rules for the session; and an opportunity to address questions, concerns, hopes, and fears related to the medication session. In the third and fourth preparation sessions (prior to the second and third drug administration sessions respectively), each topic is revisited, plans are revised based on the experience in the prior drug administration sessions, and the therapists and participant decide on the dose of medication to be used in the session within the parameters of the study protocol.

The interventions employed during the drug administration sessions are intended to help the participant use the session as productively as possible, rather than to provide directive therapy. Participants wear eyeshades and listen to a standardized program of music through headphones during most of the session. Brief check-ins are used to assess the participant's mental state, and to monitor vital signs. Therapists may provide reassurance, support, grounding, and redirection as needed. Grounding involves introducing techniques meant to help participants return to body-centered awareness and can be used to alleviate feelings of anxiety and dissociation or to facilitate return to baseline consciousness. Medications are available to treat dangerously elevated blood pressure, severe anxiety, or psychotic symptoms, but it has not yet been necessary to use any of these medications. Participants are encouraged to focus on their internal experience as much as possible, to be open and curious about whatever arises, and

actively engage with the experience by "letting go" rather than trying to direct or control it. Once the drug effects have largely subsided (after 5-6 hours) participants may spend increasing amounts of time interacting with the therapists and discussing the content and meaning of the experience. Participants are asked to write down an account of the experience during the evening after the medication sessions, for discussion at a subsequent debriefing.

A debriefing session is scheduled the day after each drug administration session. The basic content of these sessions includes open-ended inquiry concerning the drug administration session and invitation to reflect on the experience. Participants are invited to consider the meaning and implications of the experience, including any changes in views of self, relationships, values, and spirituality. Using the motivational interviewing style, therapists elicit discussion of how the session has affected the participant's relationship to alcohol and desire to change drinking behavior. See Supplemental Figure 1 for a detailed overview of the study design.

Quantitative Outcome Measures

Acute Hallucinogen Effects

The States of Consciousness Questionnaire (SCQ) is used to characterize the acute subjective effects of psilocybin treatment. This 100-item questionnaire has been used extensively to measure mystical-type states of consciousness in hallucinogen administration experiments (Griffiths, Richards, McCann, & Jesse, 2006; W. Pahnke, 1963; W. N. Pahnke, 1969b; Richards, Rhead, DiLeo, Yensen, & Kurland, 1977; Turek, Soskin, & Kurland, 1974). This scale contains the 43 items of the Pahnke-Richards Mystical Experience Questionnaire (MEQ) (Griffiths et al., 2006).

The 5-Dimensional Altered States of Consciousness Scale (5D-ASC) (Dittrich, 1998) is used to quantify acute hallucinogen effects. This scale has 94 items using the visual analog scale format, yielding 5 primary dimensions (oceanic boundlessness, anxious ego-disintegration, visionary restructuralization, acoustic alterations, and altered vigilance), and a general score (GASC).

The Hallucinogen Rating Scale (HRS), developed and validated at the University of New Mexico by Rick Strassman et al. (1994) in studies of intravenous DMT and validated in ayahuasca users (Riba, Rodriguez-Fornells, Strassman, & Barbanoj, 2001), is also administered following each drug administration session. It has been used to assess effects of a wide variety of psychoactive drugs including MDE, methamphetamine, psilocybin, ibogaine, MDMA, ayahuasca, methylphenidate, d-amphetamine, ketamine and mCPP. This 99-item scale has 6 subscales: intensity, somaesthesia, affect, perception, cognition, and volition; we report participants' scores on the intensity subscale.

Alcohol Use

The Time-line Follow-back (TLFB) (Linda C. Sobell & Sobell; L.C. Sobell & Sobell, 1996) procedure is used to assess drug and alcohol use behavior at baseline and follow-up visits. The TLFB is a semi-structured interview that provides estimates of the daily quantity, frequency, and pattern of drug and alcohol use during a specified time period. It uses a calendar prompt and number of other memory aids (e.g., holidays, payday, and other personally relevant dates) to facilitate accurate recall of drug use during the target period. The TLFB has shown adequate to excellent reliability and validity over a wide range of research and clinical contexts (Carey, 1997; L. C. Sobell, Brown, Leo, & Sobell, 1996; L. C. Sobell, Sobell, Leo, & Cancilla, 1988).

Self-efficacy

The Alcohol Abstinence Self-Efficacy Scale (AASE) (DiClemente, Carbonari, Montgomery, & Hughes, 1994) is a self-report questionnaire that has been used widely in the alcohol treatment research, both as a predictor of outcome and as a client-treatment matching variable (Project MATCH Research Group, 1997; Vielva & Iraurgi, 2001). Item content of the AASE-C subscale asks the respondent to rate the confidence they have to avoid drinking in different situations. In time-ordered analyses, a relationship has been demonstrated between the AASE-C and AA behaviors and drinking, both at 6 months (Morgenstern, Labouvie, McCrady,

Kahler, & Frey, 1997) and 3 years after completing outpatient treatment (Connors, Tonigan, & Miller, 2001).

Alcohol craving

The Penn Alcohol Craving Scale (Flannery, Volpicelli, & Pettinati, 1999) is used to assess craving. This scale has 5 Likert-scaled items with excellent internal consistency and evidence of predictive, construct, and discriminant validity.

Self-compassion

Self-compassion is a construct, derived from Buddhist psychology, which denotes a kind and non- judgmental attitude toward oneself (Neff, 2003). A reliable and valid 12-item form of the Self-Compassion Scale (Raes, Pommier, Neff, & Van Gucht, 2011) is used as measure of self-compassion.

Mood and Anxiety

The Hamilton Anxiety (Ham-A) (Hamilton, 1959) and Depression (Ham-D) (17-item) (Hamilton, 1960) scales are used as measures of depression and anxiety at baseline and at each follow-up visit.

Substance Use Consequences

The Short Inventory of Problems (SIP-2R) (W. R. Miller, Tonigan, & Longabaugh, 1995), past 3 month version, is used to measure consequences of alcohol use. The SIP-2R is a 15-item measure that assesses five domains of alcohol-related consequences: (1) social, (2) intrapersonal, (3) interpersonal, (4) impulse control, and (5) physical.

References

Bogenschutz, M. P., Forcehimes, A. A., Pommy, J. A., Wilcox, C. E., Barbosa, P. C., & Strassman, R. J. (2015). Psilocybin-assisted treatment for alcohol dependence: a proof-of-concept study. *J Psychopharmacol, 29*(3), 289-299. doi:10.1177/0269881114565144

Buckman, J. (1967). Theroetical aspects of LSD therapy. In H. A. Abramson (Ed.), *The use of LSD in psychotherapy and alcoholism* (pp. 83-100). Indianapolis: Bobbs-Merrill.

Carey, K. B. (1997). Reliability and validity of the Time-Line Follow-Back Interview among psychiatric outpatients: A preliminary report. *Psychol Addict Behav, 11*, 26-33.

Connors, G. J., Tonigan, J. S., & Miller, W. R. (2001). A longitudinal model of intake symptomatology, AA participation and outcome: retrospective study of the project MATCH outpatient and aftercare samples. *J Stud Alcohol, 62*(6), 817-825.

DiClemente, C. C., Carbonari, J. P., Montgomery, R. P., & Hughes, S. O. (1994). The Alcohol Abstinence Self-Efficacy scale. *J Stud Alcohol, 55*(2), 141-148.

Dittrich, A. (1998). The standardized psychometric assessment of altered states of consciousness (ASCs) in humans. *Pharmacopsychiatry, 31*, 80-84.

Flannery, B. A., Volpicelli, J. R., & Pettinati, H. M. (1999). Psychometric properties of the Penn Alcohol Craving Scale. *Alcohol Clin Exp Res, 23*(8), 1289-1295. doi:00000374-199908000-00001 [pii]

Griffiths, R. R., Richards, W. A., McCann, U., & Jesse, R. (2006). Psilocybin can occasion mystical-type experiences having substantial and sustained personal

meaning and spiritual significance. *Psychopharmacology (Berl), 187*(3), 268-283; discussion 284-292.

Grinspoon, L., & Balakar, J. B. (1997). *Psychedelic drugs reconsidered*. New York: The Lindesmith Center.

Grof, S. (2008). *LSD psychotherapy* (4th ed.). Ben Lomond, CA: Multidisciplinary Association for Psychedlic Studies.

Hamilton, M. (1959). The assessment of anxiety status by rating. *British Journal of Medical Psychology, 32*, 50-55.

Hamilton, M. (1960). A rating scale for depression. *J Neurol Neurosurg Psychiatry, 23*, 56-62.

Hoffer, A. (1967). A program for treatment of alcoholism: LSD, malvaria, and nicotinic acid. In H. A. Abramson (Ed.), *The use of LSD in psychotherapy and alcoholism* (pp. 343-406). Indianapolis: Bobbs-Merrill.

Leuner, H. (1967). Present state of psycholytic therapy and its possibilities. In H. A. Abramson (Ed.), *The use of LSD in psychotherapy and alcoholism* (pp. 101-116). Indianapolis: Bobbs-Merrill.

Masters, R., & Houston, J. (2000). *The Varieties of Psychedelic Experience: The Classic Guide to the Effects of LSD on the Human Psyche*. Rochester, Vermont: Park Street Press.

Miller, W. R. (Ed.) (2004). *Combined Behavioral Intervention manual: A clinical research guide for therapists treating people with alcohol abuse and dependence (Vol. 1)*. Bethesda, MD: National Institute on Alcohol Abuse and Alcoholism.

Miller, W. R., Tonigan, J. S., & Longabaugh, R. (1995). *The Drinker Inventory of Consequences (DrInC): An instrument for assessing adverse consequences of alcohol abuse. Test Manual (Vol. 4)*. Rockville, MD: US Government Printing Office.

Miller, W. R., Zweben, A., Diclemente, C. C., & Rychtarik, R. G. (1992). *Motivational enhancement therapy manual: A clinical research guide for therapists treating individuals with alcohol abuse and dependence* (Vol. 2). Rockville, MD: National Institute on Alcohol Abuse and Alcoholism.

Morgenstern, J., Labouvie, E., McCrady, B. S., Kahler, C. W., & Frey, R. M. (1997). Affiliation with Alcoholics Anonymous after treatment: a study of its therapeutic effects and mechanisms of action. *J Consult Clin Psychol, 65*(5), 768-777.

Neff, K. D. (2003). The developmen and validation of a scale to measure self-compassion. *Self and Identity, 2*, 223-250.

Obert, J. L., & Farentinos, C. (2000). *A Manual for Motivational Enhancement Treatment (MET) to Improve Treatment Engagement and Outcome in Subjects Seeking Treatment for Substance Abuse*. Manual for Protocol 0004, NIDA Clinical Trials Network.

Pahnke, W. (1963). *Drugs and mysticism: an analysis of the relationship between psychedelic drugs and the mystical consciousness.* (Ph.D.), Harvard University,

Pahnke, W. N. (1969a). Psychedelic drugs and mystical experience. *Int Psychiatry Clin, 5*(4), 149-162.

Pahnke, W. N. (1969b). Psychedelic drugs and mystical experience. *Int Psychiatry Clin, 5*, 149-162.

Project MATCH Research Group. (1997). Project MATCH secondary a priori hypotheses. *Addiction, 92*(12), 1671-1698.

Raes, F., Pommier, E., Neff, K. D., & Van Gucht, D. (2011). Construction and factorial validation of a short form of the Self-Compassion Scale. *Clin Psychol Psychother, 18*(3), 250-255. doi:10.1002/cpp.702

Riba, J., Rodriguez-Fornells, A., Strassman, R. J., & Barbanoj, M. J. (2001). Psychometric assessment of the Hallucinogen Rating Scale. *Drug Alcohol Depend, 62*(3), 215-223.

Richards, W. A., Rhead, J. C., DiLeo, F. B., Yensen, R., & Kurland, A. A. (1977). The peak experience variable in DPT-assisted psychotherapy with cancer patients. *J Psychedelic Drugs, 9*, 1-10.

Sherwood, J. N., Stolaroff, M. J., & Harman, W. W. (1962). The psychedelic experience--a new concept in psychotherapy. *J Neuropsychiatr, 4*, 69-80.

Sobell, L. C., Brown, J., Leo, G. I., & Sobell, M. B. (1996). Reliabiity of the Alcohol Timeline Followback when administered by telephone ad by computer. *Drug and Alcohol Dependence, 42*, 49-54.

Sobell, L. C., & Sobell, M. B. (1992). Timeline follow-back: A technique for assessing self-reported alcohol consumption. In R. A. Litten & J. P. Allen (Eds.), *Measuring alcohol consumption: Psychosocial and biological methods*. Totowa, NJ: Humana Press.

Sobell, L. C., & Sobell, M. B. (1996). *Timeline Follow Back: A calendar method for assessing alcohol and druq use (User's Guide).* Toronto: Addiction Research Foundation.

Sobell, L. C., Sobell, M. B., Leo, G. I., & Cancilla, A. (1988). Reliability of a timeline method: assessing normal drinkers' reports of recent drinking and a comparative evaluation across several populations. *British Journal of Addiction, 83*, 393-402.

Strassman, R. J., Qualls, C. R., Uhlenhuth, E. H., & Kellner, R. (1994). Dose-response study of N,N-dimethyltryptamine in humans. II. Subjective effects and preliminary results of a new rating scale. *Arch Gen Psychiatry, 51*(2), 98-108.

Turek, I. S., Soskin, R. A., & Kurland, A. A. (1974). Methylenedioxyamphetamine (MDA) subjective effects. *J Psychedelic Drugs, 6*, 7-14.

Vielva, I., & Iraurgi, I. (2001). Cognitive and behavioural factors as predictors of abstinence following treatment for alcohol dependence. *Addiction, 96*(2), 297-303.
